# Anti-obesity effect of *Angelica keiskei* Jiaosu prepared by yeast fermentation on high-fat diet-fed mice

**DOI:** 10.3389/fnut.2022.1079784

**Published:** 2023-01-09

**Authors:** Kunli Fu, Xiang Gao, Puyue Hua, Yuedi Huang, Ruitao Dong, Mingji Wang, Qun Li, Zichao Li

**Affiliations:** ^1^College of Life Sciences, Institute of Biomedical Engineering, Qingdao University, Qingdao, China; ^2^Anqiu Huatao Food Co., Ltd., Weifang, China; ^3^College of Chemistry and Chemical Engineering, Qingdao University, Qingdao, China; ^4^Joint Institute of Angelica keiskei Health Industry Technology, Qingdao Balanson Biotech Co., Ltd., Qingdao, China

**Keywords:** *Angelica keiskei*, Jiaosu, total flavonoids, anti-obesity, mechanism

## Abstract

In this study, an *Angelica keiskei* (*A. keiskei*) Jiaosu (FAK) was prepared by yeast fermentation to investigate its anti-obesity effect on high-fat diet (HFD)-fed mice. 70 SPF grade male C57BL/6J mice were randomly divided into 7 groups (*n* = 10): blank control group (N), high-fat model group (M), positive control group (Orl), unfermented control group (NF), high-dose intervention group (FH), medium-dose intervention group (FM), and low-dose intervention group (FL). The results showed that FAK intervention significantly reduced the body weight, Lee’s index and liver index of HFD-fed mice (*P* < 0.05). Compared with M group, the serum levels of triglyceride (TG), total cholesterol (TC), leptin and glucose (GLU) in FH group were remarkably decreased and that of interleukin-27 (IL-27) were increased (*P* < 0.05). The levels of TG, and TC in the liver of mice were also markedly decreased in the FH group (*P* < 0.05). HE staining results showed that the liver cells in the three intervention groups had less degeneration and fatty vacuoles in the cytoplasm, and the liver cords were orderly arranged compared with that of M group. Furthermore, FAK significantly inhibited epididymal adipose tissue cell expansion induced by HFD. FAK up-regulated the protein expression levels of p-AMPK and PPARα to promote lipolysis and down-regulated the expression of PPARγ to reduce lipid synthesis (*P* < 0.05). Additionally, the results of gut microbiota showed that after the intervention, a decrease trend of F/B value and *Deferribacterota* was noticed in the FH group compared with M group. At the genus level, FAK intervention significantly increased that of *Ileiobacterium* compared to the M group (*p* < 0.05). A rising trend of norank_f_*Muribaculaceae*, *Lactobacillus*, and *Bifidobacterium* were also observed in the HF group. Conclusively, these findings demonstrated that FAK intervention can effectively improve obesity in mice caused by HFD and the potential mechanisms was related to the regulation of serum levels of leptin and IL-27, lipogenesis and lipolysis in adipose tissue and gut microbiota composition.

## 1. Introduction

Over the past few decades, the global prevalence of obesity has rapidly increased, resulting in a worldwide issue (1). Obesity is a multifactorial chronic disease with multiple complications, which is associated with a variety of diseases, including metabolic diseases (e.g., type 2 diabetes), hypertension, non-alcoholic fatty liver disease, musculoskeletal diseases, cardiovascular diseases, psychiatric diseases, and more seriously, certain types of cancer. It seriously threatens human life and health, and damages the quality of life (2). At present, the change of the global dietary structure and the lifestyle of more calorie intake and less physical activity have become the main driving factors for the occurrence of obesity. Treatment for obesity now focuses on lifestyle changes (low-calorie diets and increased physical activity), drug therapy, and bariatric surgery (3). However, due to the side effects of drugs and the fact that some individuals are not suitable for surgery, dietary intervention remains the most ideal way to curb obesity.

The word “Jiaosu” was originated in Japan and first became popular in European and American countries. Until the last decade, it has begun to be recognized by the Chinese and thrived in the local market. In December 2018, the Chinese Ministry of Industry and Information Technology issued the “Guidelines for Jiaosu Products Classification” (QB/T 5324-2018), in which Jiaosu was officially defined as: a product containing specific biologically active ingredients obtained by microbial fermentation, using animals, plants, fungi, etc. as raw materials, with or without added auxiliary materials. A large number of experimental studies have proved that Jiaosu cannot only retain the original nutrients in the raw food ingredients, but also its probiotics can hydrolyze macromolecular substances, produce enzymes and increase the content of phenolic compounds, vitamins, minerals and other metabolites, thereby greatly improving bioavailability (4). Meanwhile, it has the advantages of sensory improvement, prolonging the shelf life and etc. (5). Increasing evidence indicated that Jiaosu can effectively lower body weights and alleviate obesity. Verón et al. (6) reported that the cactus pear (Opuntia ficus-indica) fruit juice fermented by autochthonous *Lactobacillus plantarum* S-811 can significantly reduce the body weights in the high-fat diet (HFD) mice, suggesting it had the potential to prevent and improve obesity and related diseases. Pan et al. examined the anti-obesity effect of fermented lemon peel on high-fat diet induced (HFD-induced) obese mice, and the results showed that the supernatant of fermented lemon peel significantly inhibited body weight gain and improved liver and epididymal adipose tissue accumulation in HFD mice lesions (7). Cho et al. found that fermented green tea extract suppressed adipogenesis and lipogenesis in adipocytes by enhancing mRNA expression of fatty acid oxidation-related genes (8).

*Angelica keiskei* (*A. keiskei*) is a perennial cold-hardy herb of the *Umbelliferae* family. It is native to the large islands of Hachijo and Izu on the Pacific coast of Japan (9). To date, it has been cultivated in Shandong, Yunnan, Guangxi, Jiangsu, and Hainan provinces etc. in China (10), and was officially recognized as a new food ingredient by the National Health Commission in 2019. *A. keiskei* is rich in chalcone, flavonoids, coumarin, and other bioactive substances (11), and shows a variety of potential biological activities, such as antitumor, antioxidant, hypoglycemic, hypotensive, liver protection, antibacterial, and antiviral, etc. (12–14). The aerial parts (stems and leaves) have been eaten for centuries as a healthy vegetable, and the whole plant can be used medicinally. Collectively, *A. keiskei* has been recognized as a valuable, healthy and green functional food source.

To our knowledge, there is no scientific report to prepare Jiaosu with *A. keiskei* as raw material and to study its anti-obesity effect *in vivo*. Herein, the *A. keiskei* Jiaosu was firstly prepared via optimized yeast fermentation, and particularly, its effects on weight loss in HFD induced obese mice were studied. Furthermore, the underlying mechanism involves the regulation of lipid synthesis and lipolysis and intestinal microbiota were investigated. The findings will provide research basis for the in-depth functional food development and high-value utilization of *A. keiskei*.

## 2. Materials and methods

### 2.1. Food ingredients and reagents

*A. keiskei* freeze-dried powder was provided by Shandong *A. keiskei* Biotech Co., Ltd. (Rizhao, China). Orlistat was purchased from Zeen Biotechnology Co., Ltd. (Changsha, China). XTHF60-1 high-fat feed was purchased from Jiangsu Cooperation Pharmaceutical Bioengineering Co., Ltd. (Nanjing, China).

### 2.2. The preparation of *A. keiskei* Jiaosu

The process parameters for preparation of *A. keiskei* Jiaosu were optimized and fixed through a series of pretests. Briefly, highly active dry yeast (*Saccharomyces cerevisiae*) and distilled water were mixed and dissolved at a ratio of 1:10 (g/mL), and activated at 37°C for 30 min. Subsequently, *A. keiskei* freeze-dried powder of 17.5 g was added to 200 mL sterile water and mixed well. Then 1.5% (v/v) activated yeast liquid was inoculated into the *A. keiskei* fermentation broth, and fermented at 37°C for 96 h. After that, the supernatant was centrifuged and freeze-dried for 24 h to obtain *A. Keiskei* Jiaosu (FAK) powder, dissolved in dosage and administered intragastrically. In addition, we also set a unfermented *A. keiskei* group, in which the *A. keiskei* freeze-dried powder was not inoculated with yeast, while the rest were the same to the processes of preparing FAK.

### 2.3. Determination of chemical constituents of FAK

The total flavonoid contents of FAK were determined by NaNO_2_-AL(NO_3_)_3_-NaOH chromogenic method as reported (13). The levels of γ-aminobutyric acid was detected by a colorimetric method (15). The contents of free amino acids was determined by ninhydrin colorimetric method. Total protein was measured by the biuret method. Total phenolic content was determined according to the Folin-Ciocalteu colorimetric method (16). The pH value was measured directly using an acidity meter. Soluble solids were measured using a hand-held food sugar meter.

### 2.4. Protocols of animal study

All animal experimental protocols and procedures were approved by the Ethics Committee of Qingdao University, and the experimental unit license number was SYXK (LU) 20200009. Seventy specific pathogen free male C57BL/6J mice [License number: SCXK (Beijing) 2019-0010] aged 6–8 weeks were purchased from SPF (Beijing) Biotechnology Co., Ltd. After a 1 week adaptively period, the mice were weighted and 10 mice were randomly selected as blank control group (N), which were kept feeding with common chow (main composition: soybean meal, fish meal, beer yeast powder, vegetable oil, bran, corn, wheat, vitamins, and minerals, and etc.) during the experimental period. The rest of the mice were fed with a HFD (32.5% basal diet, 28% lard, 8% sucrose, 10.8% whole milk powder, 13.5% casein, 3% premix for experimental animals, and 2% microcrystalline cellulose, 1.8% calcium bicarbonate, 0.4% stone powder) for 8 week for model building. Afterward, the obese model mice were randomly divided into 6 groups according to body weight (*n* = 10): the high-fat model group (M), given 10 mL/kg/d of normal saline; the positive control group (Orl), given 30 mg/kg/d orlistat; unfermented control group (NF), given 400 mg/kg/d unfermented *A. keiskei* freeze-dried powder; the high-dose intervention group (FH), given 400 mg/kg/d FAK; the middle-dose intervention group (FM), given 200 mg/kg/d FAK; the low-dose intervention group (FL), given 100 mg/kg/d FAK. The treatment doses were referenced to previous report (17). The FAK dose of 400 mg/kg/d (400 mg of FAK powder was obtained from 20 mL of Jiaosu solution after freeze-drying) was equivalent to consumption of 113.82 mL Jiaosu solution/day by a 70 kg human according to the *K*_*m*_ factor ratio of 3 and 37 for mice (30 g) and humans (70 kg), respectively. The gavage intervention was performed daily at a fixed time for 8 weeks. The body weights of mice were measured once a week. All the mice were free access to drinking and eating, and kept in the Animal Center of the Medical Department of Qingdao University, under a standard condition.

### 2.5. Blood and tissue samples acquired

Lastly, the mice in each group were fasted for 8 h, weighed, and their feces were collected and stored in a refrigerator at −80°C. After the mice were sacrificed, the blood was collected and centrifuged at 3,500 r/min for 15 min to obtain the serum. The adipose tissue of the liver and epididymis were removed, washed and weighed. After that, a portion of the liver tissues was fixed in a 4% paraformaldehyde solution for pathological analysis, and the rest of the liver tissues were immediately frozen with liquid nitrogen and stored at −80°C until analysis. The epididymal adipose tissues were fixed with a 10% formalin solution.

### 2.6. Determination of Lee’s index and liver index

The Lee’s index and liver index were calculated according to the following equation (18).


$${\mathrm{Lee}}^{{}^{\prime }}\,s\,\mathrm{index}=\frac{\sqrt[{3}]{\mathrm{Body}\,\mathrm{weigt}\;\left(g\right)*10^{3}}}{\mathrm{Mouse}\,\mathrm{length}\,\left(\mathrm{cm}\right)}$$

$$\mathrm{Liver}\,\mathrm{index}=\frac{\mathrm{Mice}\,\mathrm{liver}\,\mathrm{wet}\,\mathrm{weight}\;\left(g\right)}{\mathrm{Body}\,\mathrm{weight}\;\left(g\right)}\times 100\%$$

### 2.7. Biochemical analysis

The serum levels of triglyceride (TG), total cholesterol (TC), high-density lipoprotein cholesterol (HDL-c), low-density lipoprotein cholesterol (LDL-c), aspartate aminotransferase (AST), alanine aminotransferase (ALT), and glucose (GLU) were analyzed by an automatic biochemical analyzer (Chemray 800, Redu life science Co., Ltd., Shenzhen, China). The levels of leptin and Interleukin-27 (IL-27) in serum were detected via an ELISA kit (EK227-02, Lianke Biotechnology Co., Ltd., Hangzhou, China).

### 2.8. Histological analysis

Part of liver and epididymal tissues were fixed with 10% neutral formaldehyde, routinely embedded in paraffin, sectioned, deparaffinized, stained with hematoxylin and eosin (HE) or Oil-Red O, and sealed with neutral gum. The morphology was observed by an optical microscope (Eclipse E100, Nikon Inc., Tokyo, Japan).

### 2.9. Analysis of gut microbiota in mice

The microbial DNA in the feces were extracted and the high-throughput sequencing on the V3--V4 hypervariable regions of the bacterial 16S rRNA was performed on the Illumina MiSeq platform (Illumina, San Diego, USA) at Majorbio Bio-Pharm Technology Co., Ltd. (Shanghai, China). The data were analyzed on the free online platform.^1^

### 2.10. Western blotting analysis

Total protein was extracted from epididymal adipose tissue. The cytosolic protein lysates were prepared by lysing the cells with RIPA buffer containing 1% protease inhibitor and 1% PMSF. After centrifuging at 12,000 rpm and 4°C for 10 min, the protein concentration of the cell lysate was tested using a bicinchoninic acid (BCA) assay kit (Wanleibio Co., Ltd., Shenyang, WLA004, China) before solubilization in the loading buffer. Equal amounts of proteins were separated on SDS-PAGE and subsequently transferred to PVDF membranes. Then, the PVDF membrane was blocted in 5% milk solution before incubated with various primary antibodies (Wanleibio Co., Ltd., Shenyang, China): P-AMPK α1/2 (WL05103), PPARα (WL00978), PPARγ (WL01800), β-actin (WL01372). After that, the PVDF membranes were washed in TBST (5 min × 4 times) and combined with the secondary antibody goat anti-rabbit IgG-HRP (WLA023). The ECL luminescence solution (WLA003) was utilized for luminescence. Finally, the films were scanned and the intensities of bands were analyzed.

### 2.11. Statistical analyses

The experimental data were presented as mean ± standard deviation (SD) and the statistical analysis were performed by the Origin 2019 software. Data variability between groups was analyzed by the one-way analysis of variance (ANOVA). A *p*-value < 0.05 was accepted as statistical significance.

## 3. Results

### 3.1. Analysis of chemical ingredients composition of FAK

The optimum fermentation process for FAK was obtained via a series of preliminary experiments. Table 1 displays the chemical composition of both FAK and *A. keiskei* FD powder without fermentation (control group). As seen, comparatively, the content of free amino acids, γ-aminobutyric acid (GABA) and total flavonoids in FAK were raised by 20.68, 4.86, and 2.41 times via yeast fermentation, respectively. Moreover, the content of total chalcone, total phenolic content, protein, and pH value were all markedly increased (*P* < 0.05), while no notable change was observed in the soluble solids, indicating that the yeast fermentation process effectively elevated the active ingredient contents of *A. keiskei*, thus further improved its healthy properties for application.

**TABLE 1:** Analysis of chemical ingredients composition of FAK.

Index	Control group	FAK
Gamma-aminobutyric acid content/(g/L)	1.74 ± 0.003^a^	8.46 ± 0.09^b^
Total flavonoids/(g/L)	0.58 ± 0.08^a^	1.40 ± 0.01^b^
Total chalcone content/(g/L)	0.03 ± 0.01^a^	0.05 ± 0.01^b^
Total phenolic content/(g/L)	0.03 ± 0.02^a^	0.07 ± 0.02^b^
Free amino acid content/(g/L)	0.53 ± 0.04^a^	10.96 ± 0.64^b^
Protein content/(g/L)	2.15 ± 0.82^a^	4.11 ± 0.12^b^
pH	3.69 ± 0.10^a^	5.44 ± 0.10^b^
Soluble solids/%	3.65 ± 0.21^a^	3.63 ± 0.29^a^

^a,b^The different superscripts means significant difference statistically (*P* < 0.05).

### 3.2. Growth parameters of the mice

**FIGURE 1:**
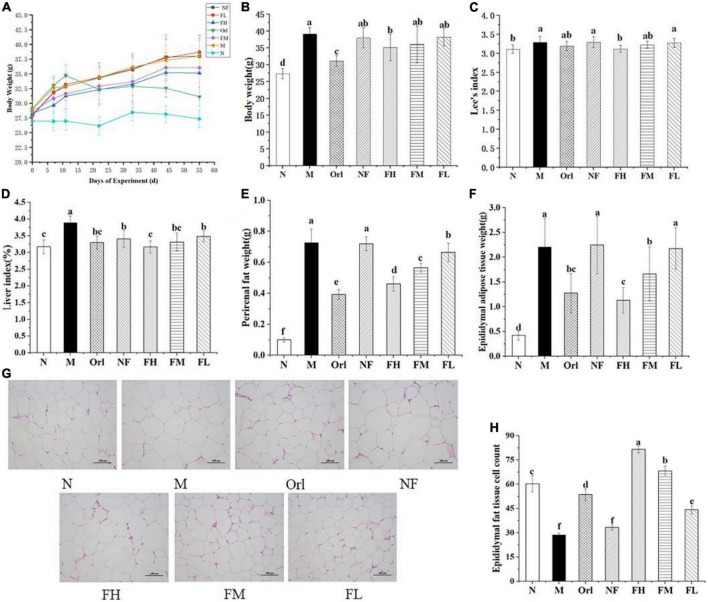
FAK ameliorates HFD-induced obesity in mice. Effects of FAK on Body weight change **(A)**; final body weight **(B)**; Lee’s index **(C)**; liver index **(D)**; perirenal fat weight **(E)**; epididymal adipose tissue weight **(F)**; H&E staining of epididymal adipose tissue (scale bar = 100 μm) **(G)**; and adipocytes count **(H)** in each group. All data are expressed as mean ± SD (*n* = 5). (a–f) Bands with different letters indicate significant differences (*P* < 0.05).

The body weight change, final body weight, Lee’s index and liver index of mice were recorded as Figures 1A–D. All mice showed similar body weight at the start of the experiment. After 8 weeks feeding, the final body weight of the mice in the M group was markedly increased by 43.35% compared with the N group (*P* < 0.05), along with increased Lee’s index and liver index (*P* < 0.05). These data suggest that obesity was successfully induced in mice by HFD feeding. Compared with the M group, the body weight of the mice in the Orl group and the FH group was notably decreased by 20.60 and 7.57%, respectively (*P* < 0.05). A dose-effect relationship was observed among the FH, FM, and FL group, while there was no prominent difference between the NF group and the M group (*P* < 0.05), indicating that the body weight of mice on a HFD can be effectively controlled by the intervention of FAK. The Lee’s index of the FH group was significantly lower than those of the M group as well (*P* < 0.05). Compared with the M group, the liver index of the Orl, FH, FM, and FL group were drastically declined by 2.97, 5.32, 2.01, and 0.52% (*P* < 0.05), respectively. Nevertheless, there was no notable difference between the N group and the FH group (*P* > 0.05).

As shown in Figures 1E,F, both the weight of perirenal and epididymal adipose tissues were significantly increased in the M group over the N group (*P* < 0.05). The weight of perirenal adipose tissue was notably decreased by the intervention of FAK and Orl (*P* < 0.05). The epididymal adipose tissue weights in the Orl, FH, and FM group were markedly declined, in comparison with those of the M group. A clear dose-effect relationship can be noticed among the FH, FM, and FL groups. No difference was found between the NF and M group (*P* > 0.05). It can be deduced that the increase in visceral fat in mice induced by HFD was effectively ameliorated by FAK supplement. The morphology of epididymal fat was further examined by HE staining (Figure 1G). The size of epididymal adipocytes in the M group was markedly larger than that in the N group. Combined with Figure 1H to measure the cells volume, it can be found that the number of cells per unit area was significantly reduced in the M group, compared with the N group (*P* < 0.05). Compared with the M group, the adipocytes per unit area in the Orl group, FH group, FM group, and FL group were increased by 87.21, 184.88, 138.37, and 54.65%, respectively (*P* < 0.05), which proved that HFD-induced enlargement of epididymal adipose tissue cells in mice was also eminently improved by FAK supplement.

### 3.3. Effects of FAK on the liver of mice

**FIGURE 2:**
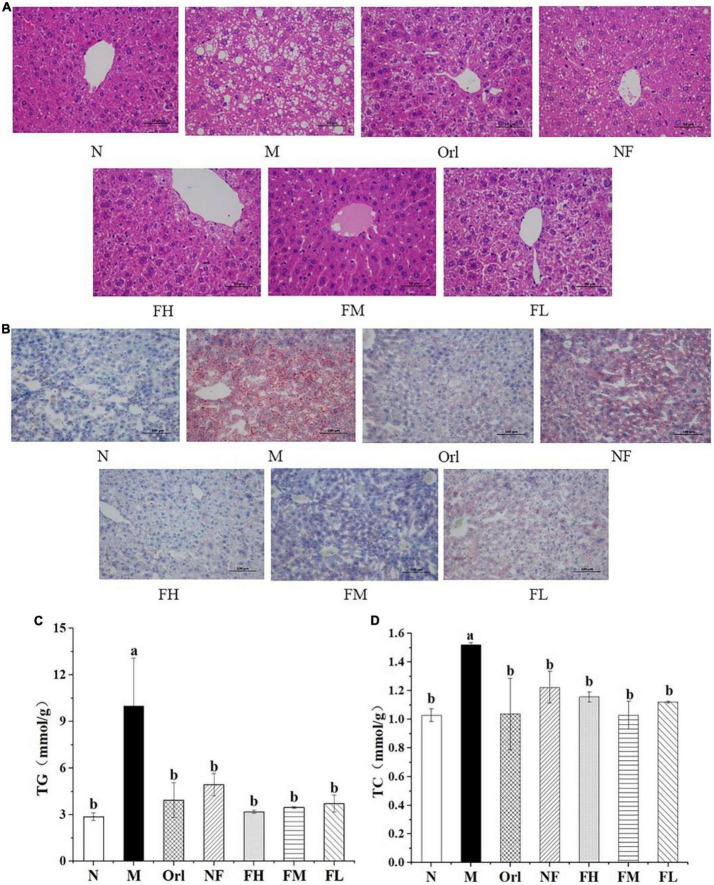
Effects of FAK on the mice liver. **(A)** H&E staining of liver (scale bar = 50 μm). **(B)** Oil-Red-O staining of liver (scale bar = 100 μm). **(C)** TG levels in liver. **(D)** TC levels in liver. All data are expressed as mean ± SD (*n* = 5). (a,b) Bands with different letters indicate significant differences (*P* < 0.05).

The morphology of liver was observed by HE and Oil Red-O staining, respectively. It can be observed from Figure 2A that the hepatocytes of the mice in the N group were relatively intact, with radial arrangement of hepatic cords and abundant cytoplasm, and their nuclei were located in the center of the cells with clear boundaries. The cytoplasm of liver cells of mice in the M group was filled with a large number of small fat vacuoles, the cell morphology was destroyed, the arrangement of liver cords was disordered, and the cell boundaries were blurred. Compared with the M group, the degeneration of liver cells in the Orl, NF, FH, FM, and FL group were improved, the fat vacuoles in the cytoplasm were reduced, the liver cords were neatly arranged, and the cell morphology was relatively intact, while the improving effect of the NF group was not ideal.

As shown in Figure 2B, there were more red areas stained by Oil Red O staining in the M group than the N group. Among all the experimental groups, the intracellular lipid content of the FH group was comparable to that of the N group, of which the improving effect was the best, although the fat accumulations in the liver cells for the Orl, FM, and FL group were also improved.

The levels of TG and TC in liver of mice were also determined, as shown in Figures 2C,D. Compared with the N group, the levels of TG and TC in the mice liver for the M group were significantly raised by 248.93 and 48.03% (*P* < 0.05), respectively. Compared with the M group, the TG and TC levels of the other groups were markedly reduced (*P* < 0.05), indicating that the accumulation of hepatic fat caused by HFD in mice was effectively ameliorated by FAK intervention.

### 3.4. Effects of FAK on serum biochemicals of mice

**FIGURE 3:**
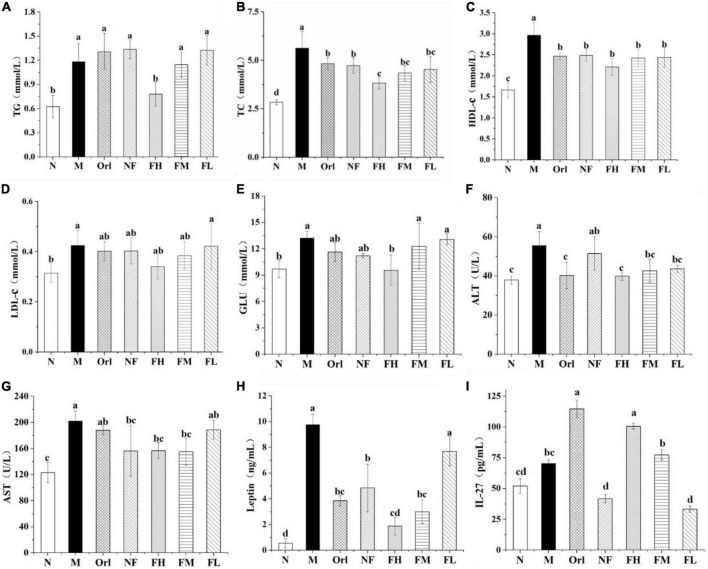
Effects of FAK on serum levels of TG **(A)**; TC **(B)**; HDL-c **(C)**; LDL-c **(D)**; GLU **(E)**; ALT **(F)**; AST **(G)**; leptin **(H);** and IL-27 **(I)** in mice. All data are expressed as mean ± SD (*n* = 5). (a–d) Bands with different letters indicate significant differences (*P* < 0.05).

Figures 3A–D shows the levels for serum biochemicals of mice. The serum TG, TC, and LDL-c of the M group mice were dramatically elevated than those of the N group (*P* < 0.05). Compared with the M group, serum TG levels in the FH and FM group were markedly decreased by 46.51 and 21.55%, respectively (*P* < 0.05). The levels of serum TC in the Orl, NF, FH, FM, and FL group were prominently declined by 14.09, 15.86, 31.92, 22.71, and 19.44%, respectively (*P* < 0.05). The serum HDL-c contents of the M group were increased by 78.45%, compared with those of the N group (*P* < 0.05), while there was no remarkable distinction among the other experimental groups (*P* > 0.05). Moreover, that the serum glucose levels of the mice (Figure 3E) in the M group was notably higher than that in the N group (*P* < 0.05). Compared with the M group, the serum glucose levels in the FH group was significantly decreased (*P* < 0.05). There was no conspicuous difference between the other experimental groups and the M group (*P* > 0.05). As seen in Figures 3F,G, the serum ALT and AST levels of the M group was markedly elevated by 46.62% (*P* < 0.05) than the N group. Compared with the M group, the Orl and FAK treated groups exhibited significant declined ALT levels (*P* < 0.05). There was no distinction in ALT levels between NF and M groups (*P* > 0.05). The levels of AST in the serum of the Orl, FH, and FM group were eminently declined (*P* < 0.05) by 7.10, 22.79, and 22.45%, respectively.

We also detected serum levels of leptin and IL-27 of the mice. As shown in Figures 3H,I, the serum leptin levels of the mice in the M group was 17.59 times that of the N group. Compared with the M group, the serum leptin levels of the Orl, NF, FH, and FM groups were markedly decreased (*P* < 0.05) by 60.52, 50.28, 80.68, and 69.26%, respectively. The serum levels of IL-27 in each group are shown in Figure 3F. Compared with the M group, the serum IL-27 levels of the Orl and FH group were raised by 116.83 and 94.35%, respectively (*P* < 0.05), and a dose-effect relationship was noticed on the FAK treated groups.

### 3.5. Effects of FAK on the expression of genes related to lipid metabolism in epididymal adipose tissue

**FIGURE 4:**
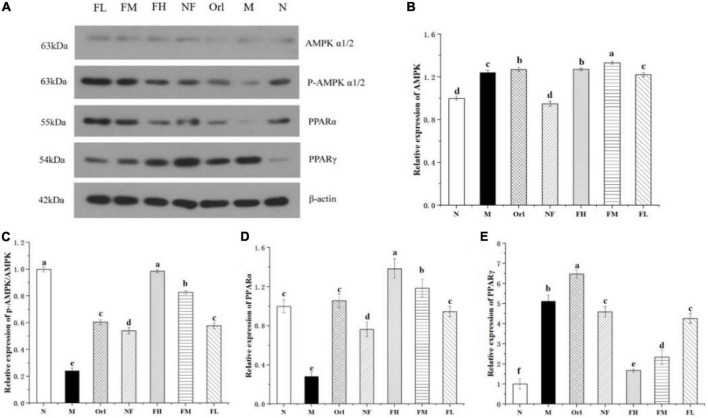
Effects of FAK on the epididymal adipose tissue in mice. **(A)** Bands of WB assay. **(B–E)** Relative protein expression of corresponding genes. All data are expressed as mean ± SD (*n* = 5). (a–d) Bands with different letters indicate significant differences (*P* < 0.05).

To explore the potential mechanisms underlying the anti-obesity effect of FAK, we measured the expression of several proteins related to lipid metabolism in epididymal adipose tissue (Figures 4A–E). The results show that, compared with the N group, the relative expressions of p-AMPK/AMPK and PPARα in the M group were significantly reduced (*P* < 0.05), and that of PPARγ was markedly elevated (*P* < 0.05). Compared with the M group, the relative expressions of p-AMPK/AMPK and PPARα in the FH, FM, and FL groups were remarkably raised (*P* < 0.05), and that of PPARγ was notably declined (*P* < 0.05). Moreover, a dose-response relationship was found among the FH, FM, and FL groups. Therefore, it can be inferred that FAK improved HFD-induced obesity in mice by regulating the expressions of lipid metabolism-related proteins.

### 3.6. Effects of FAK on the gut microbiota of mice

**FIGURE 5:**
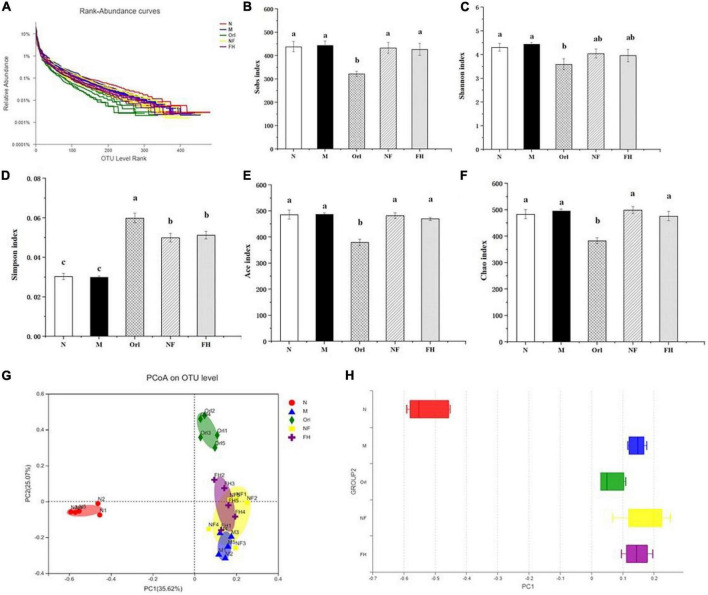
Effects of FAK on gut microbes in mice. **(A)** Rank-Abundance curves. **(B–F)** Sobs, Shannon, Simpson, Ace, and Chao indices. **(G)** PCoA analysis. **(H)** Statistical analysis of PCoA. All data are expressed as mean ± SD (*n* = 5). (a–c) Bands with different letters indicate significant differences (*P* < 0.05).

The Rank-Abundance curve is mainly used to show the richness of the samples. In the Rank-Abundance curve, the smoother the curve declines, the higher the sample species diversity. As shown in Figure 5A, the mice in the N group possessed the most abundant gut microbiota species, while the Orl group had the least species of bacteria. The species of gut microbiota in the M group was lower than that in the N group, indicating that HFD can decrease the abundance of gut microbiota. The results of Alpha diversity analysis are listed in Figures 5B–F. Among them, Sobs, Ace, and Chao indices reflect community richness, while Shannon and Simpson indices show community diversity. The greater the Shannon value, the higher the community diversity. Conversely, the smaller the Simpson Index, the higher the community diversity. Compared with the M group, the Sobs, Shannon, Ace, and Chao values of the FH group were not changed, while these indexes of the Orl group were significantly decreased. FH, NF, and Orl groups exhibited higher Simpson index than the M group (*p* < 0.05). The results indicate that FAK intervention imposed no side effects on the richness and abundance of gut microbiota in the HFD mice. To observe the differences in the overall structure of gut microbiota, principal coordinate analysis (PCoA) was performed. As shown in Figures 5G,H, the HFD fed mice were significantly separated from those fed a normal diet, suggesting differences in the number of gut microbes between the M and N groups after the HFD intervention. There was also a trend of isolation between the FH group and M group, indicating that the FAK intervention markedly changed the overall structure of the gut microbiota in the HFD mice, reflecting that the intervention had a significant effect on the structure of the gut microbiota.

**FIGURE 6:**
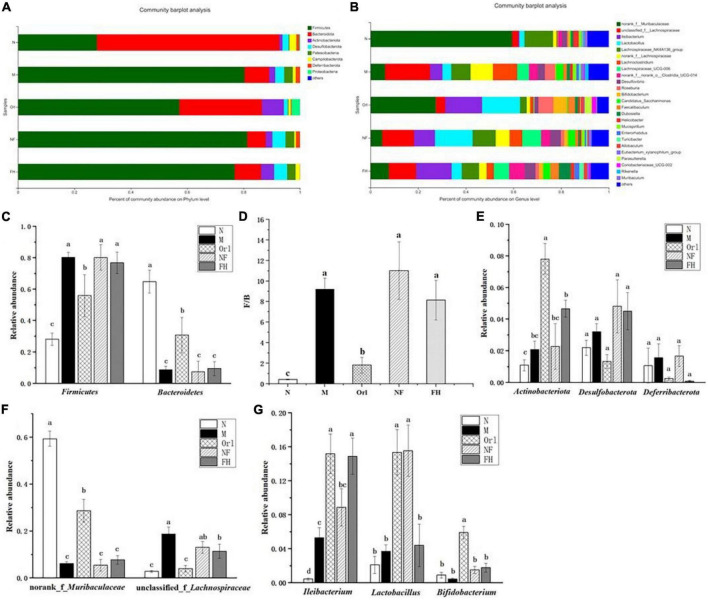
Effects of FAK on gut microbes in mice. **(A)** Relative abundance at the gut microbial phylum level. **(B)** Relative abundance at the gut microbial genus level. **(C–E)** Relative abundance of gut microbiota at the phylum level. **(F,G)** Relative abundance of gut microbiota at the genus level. All data are expressed as mean ± SD (*n* = 5). (a–c) Bands with different letters indicate significant differences (*P* < 0.05).

**FIGURE 7:**
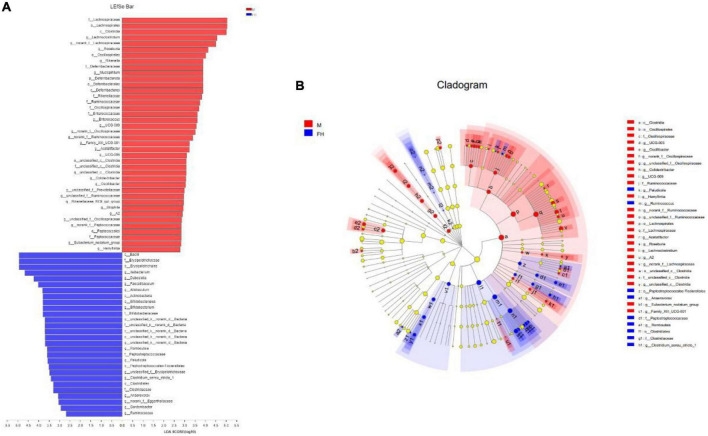
LEfSe and LDA analyses based on OTUs characterized the microbiomes of the M and FH groups. **(A)** LDA scores show the significant bacterial differences between the FH and M (log LDA > 2.0; *n* = 5). **(B)** The branching diagram shows the phylogenetic distribution of bacterial lineages in the FAK intervention group and the model group. Different colored areas represent different components (red, M; blue, FH). Circles indicate phylogenetic levels from domain to genus. The diameter of each circle is proportional to the abundance of the group.

The composition of the gut microbiota at the phylum level was displayed in Figures 6A,C–E. It can be observed that the gut microbiota of mice was mainly composed of *Firmicutes*, *Bacteroidota*, and *Actinobacteriota*. The ratio of *Firmicutes*/*Bacteroidota* (F/B) in the M group was higher than the N group. FH and Orl administration decreased the F/B value, compared with the M group. While, NF group showed a higher F/B value than the M group. HFD feeding led to an elevated trend of *Actinobacteriota*, *Desulfobacterota*, and *Deferribacterota* than the N group, while no significant difference was observed. FAK intervention significantly increased the relative levels of *Actinobacteriota* (*p* < 0.05) and led to a notable declining trend of *Deferribacterota*, compared to the M group. Orl group displayed higher relative abundance of *Actinobacteriota* than the M group (*p* < 0.05). The relative abundance of gut microbiota at the genus levels are shown in Figures 6B,F,G. As seen, the relative abundance of norank_f_*Muribaculaceae* in the M group was markedly decreased (*p* < 0.05), while unclassified_f_*Lachnospiraceae* and *Ilebacterium* were increased notably, compared with the N group (*p* < 0.05). A rising trend of *Lactobacillus* and decrease of *Bifidobacterium* were also noticed, compared to the N group. FAK intervention remarkably raised that of *Ileiobacterium*, compared to the M group (*p* < 0.05). Moreover, decline of unclassified_f_*Lachnospiraceae* and increase of norank_f_*Muribaculaceae*, *Lactobacillus* and *Bifidobacterium* were observed in the HF group as well. Meanwhile, Orl treatment enhanced the relative levels of norank_f_*Muribaculaceae*, *Ileiobacterium*, *Lactobacillus*, and decreased that of unclassified_f_*Lachnospiraceae*, compared to the M group (*p* < 0.05). However, there was a prominent increase of *Lactobacillus* in the NF group, comparing with the M group. In addition to α and β diversity analysis of the gut microbiota (19), to identify specific bacteria in the M and FH groups, gut microbiota-diverse species were analyzed using linear discriminant analysis (LDA) effect size (LEfSe) based on the non-parametric factorial KW and rank tests (20). As shown in Figure 7, by LEfSe analysis (LDA threshold of 2), c_*Bacilli*, f_*Erysipelotrichaceae*, o_*Erysipelotrichales*, and g_*Ileibacterium* were significantly enriched in the FH group compared with those in the M group, while f_*Lachnospiraceae*, o_*Lachnospirales*, and c_*Clostridia* were enriched in the M group.

## 4. Discussion

Obesity is a chronic metabolic disease that is charactered by excess accumulation of body fat in the body and has a serious negative impact on human health. In this study, *A. keiskei* was utilized as raw material for the first time to prepare Jiaosu via yeast fermentation and its anti-obesity effect was evaluated. The results showed that the contents of total flavonoid, γ-aminobutyric acid, chalcone, total phenol, free amino acids and protein of FAK were all dramatically enriched than the unfermented *A. keiskei*, indicating that the fermentation of yeast has a notably positive effect on the preparation of FAK, which may be attributed to the synergistic action of functional enzymes such as protease, cellulase, and pectinase secreted by yeast during the fermentation process. These enzymes decomposed substances such as macromolecular polysaccharides, proteins, and lipids that were difficult to be digested in plant cells into small molecules that can be easily absorbed by animals (21). Moreover, the microbial activity in the fermentation broth can also break the plant cells, thus accelerate the dissolution of phenols, flavonoids and other substances in the cells, and increase the yield. Moreover, in a high-fat induce obese mouse model, FAK effectively reduced the body weights gain, blood lipid levels, damage, and fat accumulation in liver. We also proposed the underlying mechanism in the inhibition of the expressions for the obesity-related genes and regulation of gut microbiota.

Body weight and Lee’s index can reflect the body shape of mice and are important indicators of the obesity degree in mice, and increase in liver index is often accompanied by elevation in hepatic steatosis (22, 23). Long-term intake of HFD will aggravate the expansion of adipose tissue, cause endocrine dysfunction, interfere with normal adipokines secretion and may also remodel adipose tissue by increasing the size of adipocytes. These disturbances are positively associated with metabolic disorders such as obesity, type 2 diabetes, and non-alcoholic fatty liver disease (24). In this study, both FAK and orlistat effectively reduced the weight of visceral (perirenal and epididymal) adipose tissue in mice, and the effect of FAK was proportional to the intervention dose. These results suggested that FAK had a good weight loss effect in obese mice. Moreover, FAK but not NF significantly inhibited the hypertrophy of adipocytes suggesting the fermentation process enhanced the anti-obesity effect of *A. keiskei*. It has been reported that prophylactic removal of visceral adipose tissue can prominently improve obesity-induced insulin resistance and hepatic steatosis (25). Therefore, we speculate that FAK can improve the obesity-related metabolic disorders in mice by reducing visceral adipose tissue. The components of FAK are complex and the main ingredients are total flavonoids and GABA. Zhou et al. (26) reported that GABA-rich rice bran eminently decreased the body weight and blood lipid levels of HFD-fed rats. Zhang et al. demonstrated that two major flavonoids, 4-hydroxyderricin (4-HD) and xanthoangelol (XAG) from *A. keiskei* inhibit adipocyte differentiation through the AMPK and MAPK pathways (27). The findings of Zhang et al. suggested that ingestion of flavonoid enriched fresh *A. keiskei* juice, which can prevent HFD-induced obesity and metabolic disorders by improving the intestinal microbiota composition (17). Our findings are consistent with these reports. In addition, the fermentation process also produces free amino acids, active peptides, polysaccharides, and other substances. Wang et al. employed the HPLC-MS method to prove that the components of fermented chili powder (FMPs) were transformed during fermentation, and FMPs had the higher quantity of free amino acids (28). The results of Liu et al. indicated that fermentation enhanced the anti-obesity effect of pepper on the HFD mice (29). The enriched ingredients during the fermentation process may explain the superior anti-obesity effect of FAK than NF. The liver is important for fat lipid metabolism, and the imbalance between lipid synthesis and decomposition can lead to excessive fat deposition in liver, namely fatty liver (30). The serum ALT and AST levels are potential markers for the function of liver (31). Our study showed that FAK intervention effectively improved liver function, and reduced fat accumulation in liver tissue. Lee et al. (32) demonstrated that isopsoralen chalcone isolated from *A. keiskei* reduced hepatic fat deposition caused by high-fat cholesterol diet and alleviate hepatic steatosis. Several other studies have found that GABA could effectively protect ethanol-induced and dietary-induced hepatocyte injury (33, 34). The above studies have supported that FAK enriched substances can ameliorate hepatic steatosis and protect liver form dysfunction.

The precisely mechanism of obesity is largely unknown. Previous studies indicated that various hormones involved in its process. Leptin is a main signal of energy storage and has a negative feedback effect on energy intake (35, 36). However, HFD can induce leptin resistance and lead to obesity resulting in increased blood leptin levels (37, 38). In our study, FAK treatment effectively reduced serum levels of leptin, indicated the improved leptin resistance after FAK intervention. IL-27 is a heterodimeric cytokine that regulates the immune system, promotes adipocyte browning and thermogenesis to reduce obesity symptoms (39). IL-27 could directly target adipocytes to activate p38 MAPK-PGC-1α signaling and stimulate UCP1 production, which in turn upregulates PPARα expression and promotes fat oxidation (39, 40). Herein, FAK dramatically increased the serum level of IL-27 in HFD-fed mice, suggesting the potential of promoting the burning of adipose tissue and fat oxidation in mice to ameliorate obesity. Taken together, the results indicated that the protective effect of FAK on obesity may be related to the regulation of Leptin and IL-27.

Obesity or excessive expansion of fat mass in the host mainly attribute to the unbalance between lipogenesis and lipolysis in adipose tissue. Lipogenesis involves in the synthesis of TGs and the terminal differentiation of preadipocytes into mature adipocytes. This process is tightly regulated by several key lipogenic transcription factors, such as PPARγ and SREBP-1C, that regulate a variety of signaling molecules, like FAS, ACC, etc. (41). PPARα is a key ligand-activated transcription factor that mediated lipolysis (42). AMPK is a central regulator of energy balance. When energy intake is excessive, AMPK is activated by phosphorylation that promote fat and glucose metabolism to maintain homeostasis (43). Upon AMPK activation, it controls its downstream signals, such as PPARα, UCP1 to promote lipolysis and fat oxidation (44, 45). In this study, we found that FAK up-regulated the expression of p-AMPK and PPARα protein and decreased PPARγ in adipose tissue of HFD mice, indicated the activation of lipolysis and inhibition of lipogenesis. Zhang et al. found that *A. keiskei* extract prevented obesity by regulating phosphorylation of AMPK in adipose tissue and liver (46). Another study confirmed that the natural flavonoid pre-treatment had an alleviative effect on alcoholic liver injury in mice, and the mechanism might be related to the regulation of PPARα-mediated gene expression (47). Our data agree well with these results. Therefore, we suppose that FAK protected the mice from obesity through inhibiting fat synthesis and promoting lipolysis in adipose tissue.

Increasing evidence pointed that the gut microbiota plays primary roles in modulating host metabolism and health. Diet is considered to be the most important external factor affecting the homeostasis of gut microbiota distribution (48). Previous studies have shown that obesity is tightly associated with the gut microbiota composition and the microbial diversity. Our data show that both FAK and orlistat significantly alleviated obesity and improved the related biochemical indicators. However, the diversity of intestinal flora in the Orl group was lower than that in the FH group. The reason may be that orlistat inhibited the activity of pancreatic lipase in the intestine and also affected the gut microbiota of mice (49), while FAK showed no harmful effect on the diversity of gut microbiota. At the phylum level, *Firmicutes* and *Bacteroidetes* are important categories in the intestinal flora, thus F/B (*Firmicutes*/*Bacteroidetes*, F/B) value is recognized as one of the prominent markers to evaluate obesity (50). Studies have shown that various specific gut bacteria can regulate intestinal permeability and inflammatory status of host to alleviate obesity-related metabolic syndrome (51). *Firmicutes* and *Bacteroidetes* are the most abundant phyla of humans and mice, and an elevated F/B value are considered as a hallmark of obesity (52). *Bacteroidetes* fermented dietary fibers to produce short-chain fatty acid acids (SCFAs) that is beneficial for health (53). *Actinobacteriota* was reported to be enriched in the feces of obese rodents (54). *Desulfobacterota* contributes to obesity and its complications through the production of endotoxin lipopolysaccharide (55). *Deferribacterota* in the gut is associated with inflammation and development of obesity of the host (55–57). Herein, FAK intervention led to a decrease trend of *Firmicutes*, *Deferribacterota*, and the F/B values in HFD mice, which were complied with the amelioration of obesity. At the genus level, norank_f_*Muribaculaceae* is SCFAs producing bacteria and was significantly reduced in HFD-fed mice (58). Lactobacillus and *Bifidobacterium* are probiotics and are effective in reducing obesity and controlling metabolic disorders (59, 60). *Bifidobacterium* can also reduce metabolic endotoxemia by participating in the inhibition of pathogenic adhesion to the intestinal mucosa (61), stimulate bile acid biotransformation, promote fat digestion and influence lipids Metabolism (62). Lefse with LDA was employed to estimate the effect of each species abundance on the variance. The bacteria affecting the FH group were diverse and distributed in different taxa, such as c_*Bacilli* at the phylum level and f_*Erysipelotrichaceae*, o_*Erysipelotrichales*, and g_*Ileibacterium* at the genus level. Microbial diversity makes the gut microbiota more resilient (20). In our study, the relative abundances of norank_f_*Muribaculaceae*, *Ileibacterium*, *Lactobacillus*, and *Bifidobacterium* were increased after FAK intervention, indicating the beneficial effect of FAK on gut microbiota. Collectively, it can be speculated that FAK might attenuate obesity by protecting the gut microbiota diversity and improving the gut microbiota composition.

## 5. Conclusion

**FIGURE 8:**
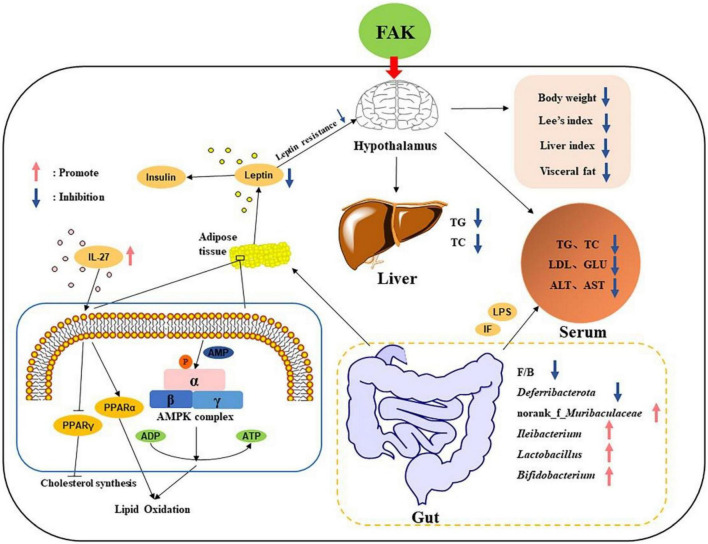
Potential mechanisms by which FAK ameliorates HFD-induced obesity.

In conclusion, our study shows that FAK can effectively ameliorate HFD-induced obesity, hyperlipemia, hepatic lipids accumulation in mice. The underlying mechanism may be related to the regulation of serum levels of leptin and IL-27, lipogenesis and lipolysis in adipose tissue and gut microbiota composition (Figure 8). This work provides key evidence that FAK prepared by yeast fermentation shows promising anti-obesity effects which can be expected to serve as a potential anti-obesity functional food.

## Data availability statement

The data presented in the study have been deposited in the SRA repository, accession number PRJNA914722 (www.ncbi.nlm.nih.gov/sra/PRJNA914722).

## Ethics statement

The animal study was reviewed and approved by the Ethics Committee of Qingdao University [SYXK (LU) 20200009].

## Author contributions

KF and XG: investigation, data curation, formal analysis, methodology, software, visualization, and writing—original draft. XG: data curation, resources, validation, and writing—review and editing. PH: investigation, formal analysis, and methodology. YH: formal analysis and visualization. RD: formal analysis. MW: funding acquisition. QL: funding acquisition and validation. ZL: conceptualization, funding acquisition, methodology, project administration, resources, supervision, validation, and writing—review and editing. All authors contributed to the article and approved the submitted version.

## Footnotes

1
https://cloud.majorbio.com
